# BrainLossNet: a fast, accurate and robust method to estimate brain volume loss from longitudinal MRI

**DOI:** 10.1007/s11548-024-03201-3

**Published:** 2024-06-16

**Authors:** Roland Opfer, Julia Krüger, Thomas Buddenkotte, Lothar Spies, Finn Behrendt, Sven Schippling, Ralph Buchert

**Affiliations:** 1 Jung Diagnostics GmbH, Hamburg, Germany; 2 Department of Diagnostic and Interventional Radiology and Nuclear Medicine, University Medical Center Hamburg-Eppendorf, Martinistr. 52, 20246 Hamburg, Germany; 3 Institute of Medical Technology and Intelligent Systems, Hamburg University of Technology, Hamburg, Germany; 4 Multimodal Imaging in Neuroimmunological Diseases (MINDS), University of Zurich, Zurich, Switzerland; 5 Neuroscience and Rare Diseases (NRD), Roche Pharma Research and Early Development (pRED), Basel, Switzerland

**Keywords:** Magnetic resonance imaging, Brain volume loss, Convolutional neural network, Multiple sclerosis

## Abstract

**Purpose:**

MRI-derived brain volume loss (BVL) is widely used as neurodegeneration marker. SIENA is state-of-the-art for BVL measurement, but limited by long computation time. Here we propose “BrainLossNet”, a convolutional neural network (CNN)-based method for BVL-estimation.

**Methods:**

BrainLossNet uses CNN-based non-linear registration of baseline(BL)/follow-up(FU) 3D-T1w-MRI pairs. BVL is computed by non-linear registration of brain parenchyma masks segmented in the BL/FU scans. The BVL estimate is corrected for image distortions using the apparent volume change of the total intracranial volume. BrainLossNet was trained on 1525 BL/FU pairs from 83 scanners. Agreement between BrainLossNet and SIENA was assessed in 225 BL/FU pairs from 94 MS patients acquired with a single scanner and 268 BL/FU pairs from 52 scanners acquired for various indications. Robustness to short-term variability of 3D-T1w-MRI was compared in 354 BL/FU pairs from a single healthy men acquired in the same session without repositioning with 116 scanners (Frequently-Traveling-Human-Phantom dataset, FTHP).

**Results:**

Processing time of BrainLossNet was 2–3 min. The median [interquartile range] of the SIENA-BrainLossNet BVL difference was 0.10% [− 0.18%, 0.35%] in the MS dataset, 0.08% [− 0.14%, 0.28%] in the various indications dataset. The distribution of apparent BVL in the FTHP dataset was narrower with BrainLossNet (*p* = 0.036; 95th percentile: 0.20% vs 0.32%).

**Conclusion:**

BrainLossNet on average provides the same BVL estimates as SIENA, but it is significantly more robust, probably due to its built-in distortion correction. Processing time of 2–3 min makes BrainLossNet suitable for clinical routine. This can pave the way for widespread clinical use of BVL estimation from intra-scanner BL/FU pairs.

**Supplementary Information:**

The online version contains supplementary material available at 10.1007/s11548-024-03201-3.

## Introduction

Whole brain volume loss (BVL) estimated from two MRI scans of the same individual, a baseline (BL) and a follow-up (FU) scan, is an useful neurodegeneration marker in chronic neurological and psychiatric conditions [1, 2]. In multiple sclerosis (MS), MRI-based BVL is associated with disability and the risk of conversion from clinically isolated syndrome to definite MS [3]. It has been proposed as a complementary marker to assess treatment response in MS [4].

SIENA (Structural Image Evaluation using Normalization of Atrophy) [5] is the state-of-the-art tool for MRI-based BVL estimation [3, 6]. It has been very well validated, including detailed characterization regarding measurement error and physiological short-term test–retest variability [7, 8]. Age-dependent thresholds on SIENA BVL estimates have been established to discriminate disease-related BVL from healthy aging [9, 10]. The in-depth validation of SIENA is the basis for its use to measure BVL as primary or secondary endpoint in phase II/III MS drug trials [11, 12].

SIENA primarily relies on brain surface changes, which limits its ability to measure regional BVL. Jacobian determinant integration leverage (JI) methods overcome this limitation [13, 14]. They are based on high-dimensional non-linear image registration of BL/FU pairs. Regional volume changes are obtained by integrating the Jacobian determinant of the deformation field across the region-of-interest. JI gained widespread acceptance for measuring regional BVL [3, 14].

SIENA and JI are computationally demanding: typical computation time per BL/FU pair is about 1 h on a standard workstation. While this is not a limitation in research, it restricts their use in clinical routine. For seamless integration into the radiologist's workflow, the volumetric analyzes must not delay report-writing [15].

SIENA and JI are built on conventional image analysis techniques. Thus, each case has to go through the entire processing pipeline. In contrast, deep learning-based approaches can be very expensive in terms of computation time for training and validation, but once this is completed, processing of new cases is fast. Thus, deep learning can drastically reduce computation time for complex image analyzes and, therefore, support their integration into busy clinical workflows. Furthermore, deep learning-based methods provide superior performance compared to conventional techniques in many image analysis tasks [16, 17]. For example, CNN-based non-linear image registration [18, 19] outperforms conventional methods including ANTS [20] and DARTEL [21].

Against this background, the current study designed, trained and tested a novel CNN-based method, “BrainLossNet”, for estimating BVL from intra-scanner T1-weighted 3D-MRI BL/FU pairs with the same accuracy as SIENA, but improved robustness and ≤ 5 min processing time.

## Materials and methods

### Datasets

All datasets comprised 3D gradient echo brain T1w-MRI scans acquired with sequences recommended by the scanner manufacturer. FU scans were acquired with the same scanner and the same sequence as the corresponding BL scan. The datasets were not curated manually. A summary of the datasets is given in Table 1.

**Table 1:** Datasets

	Training	Testing		
	Development dataset	Development dataset	MS patients	FTHP dataset
No. BL/FU pairs	1525	268	225	354
No. different subjects	1525	268	94	1
Age at BL in years, mean [range]	46.9 [18.1, 88.8]	46.2 [19.4, 83.1]	34.2 [20.1, 54.7]	49.6 [48.6, 51.2]
Delay between BL/FU pairs in years, mean (std)	1.9 (1.2)	1.9 (1.4)	1.1 (0.5)	< 1 min
No. different scanners	83	52	1	116
No. different scanner models	32	26	1	30

#### Development dataset

The development dataset was included retrospectively from a large dataset of MRI acquired in clinical routine for various indications. The only inclusion criterion was a minimum time interval of 6 months between BL and FU scan. This resulted in the inclusion of 1793 BL/FU pairs from 1793 different patients acquired with 83 different MRI scanners (32 different scanner models, Supplementary section “MR scanner models in the training dataset”).

The data of the development dataset had been provided to jung-diagnostics GmbH under the terms of the European Data Protection Regulation for remote image analysis. Subsequently, the data had been anonymized. The need for written informed consent for the retrospective use of the anonymized data was waived by the ethics review board of the general medical council of the state of Hamburg, Germany (reference number 2021-300047-WF).

The data was randomly split into 1525 (85%) BL/FU pairs for training and 268 (15%) BL/FU pairs for testing.

#### Multiple sclerosis (MS) dataset

The MS dataset retrospectively included 94 patients with confirmed MS diagnosis from an observational study at the University Hospital of Zurich, Switzerland [22]. On average, 3.5 scans were available per patient. BL/FU pairs were built from consecutive scans (for example, 3 BL/FU pairs were built for a patient with 4 scans: first/second, second/third, third/fourth). This resulted in a total of 225 BL/FU pairs. Mean BL-to-FU time interval was 1.1 ± 0.5*y*.

The retrospective use of the data was approved by the ethics committee of the Canton of Zurich, Switzerland (reference numbers 2013-0001, 2020-01187).

#### Frequently traveling human phantom (FTHP) dataset

The FTHP dataset was recently introduced by our group [23]. It comprises 557 3D-T1w-MRI scans of a single healthy male who completed 157 imaging sessions on 116 different MRI scanners within about 2.5y. The current study included all imaging sessions that comprised at least two back-to-back repeat scans that had been acquired consecutively (delay < 1 min) with the same sequence in the same session without repositioning (123 sessions on 116 different MRI scanners).

Most imaging sessions comprised three to five back-to-back scans. BL/FU pairs were built from consecutive scans within a session. This resulted in a total of 354 BL/FU pairs. BL/FU pairs combining scans from different imaging sessions were not considered. The rationale for this was that the FTHP dataset was used to assess the stability of BVL estimates with respect to short-term test–retest variability of 3D-T1w-MRI. For this purpose, the BL/FU pairs were intended to combine scans as similar as possible, that is, no volume change from BL to FU, neither true volume change associated with aging or physiological status nor apparent volume change due to scanner-/sequence-dependent variability.

The healthy individual had given written informed consent for the retrospective use of the dataset. This was approved by the ethics review board of the general medical council of the state of Hamburg, Germany (reference number PV5930). The FTHP dataset was made freely available for research purposes by our group at https://www.kaggle.com/datasets/ukeppendorf/frequently-traveling-human-phantom-fthp-dataset.

### SIENA

For benchmarking, BVL estimates were computed with SIENA (version 2.6) [5], which is part of the FMRIB Software Library (FSL; http://www.fmrib.ox.ac.uk/fsl). Parameter settings for SIENA were as described previously [7, 9]. SIENA BVL estimates were computed for all BL/FU pairs in all datasets.

### BrainLossNet

**Fig. 1:**
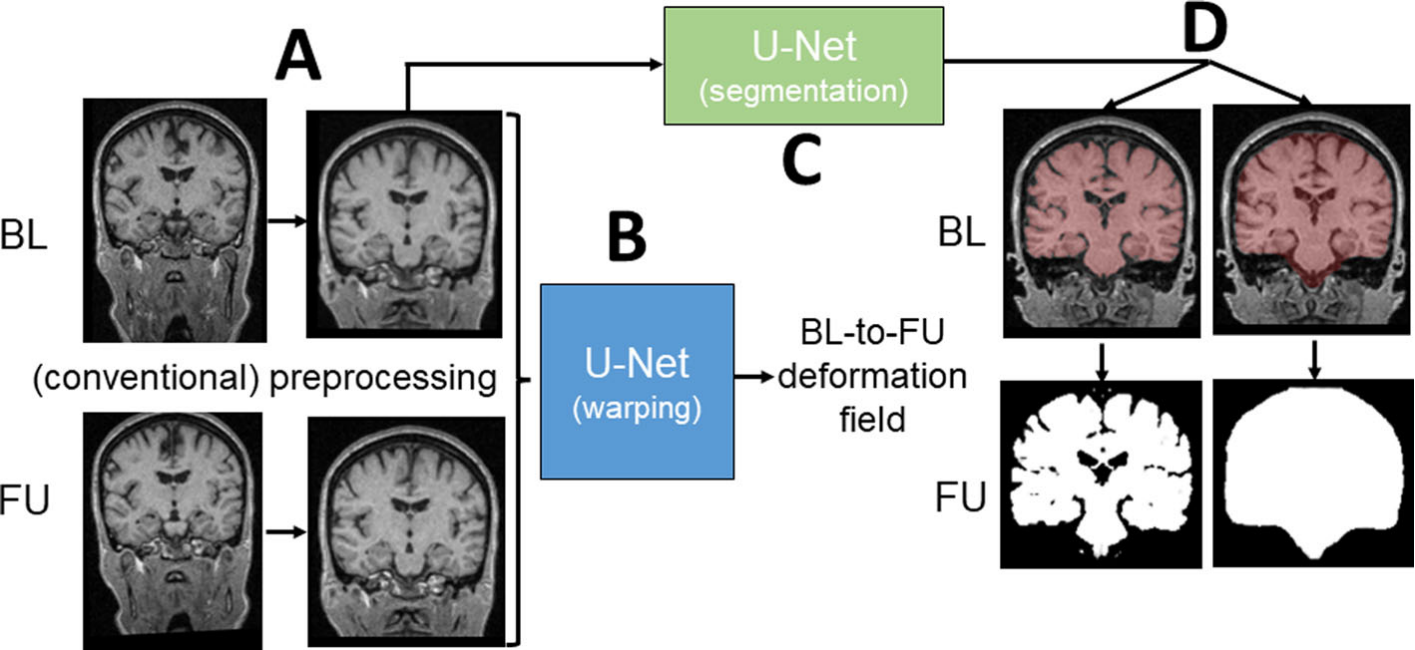
BrainLossNet pipeline

The BrainLossNet pipeline was specifically designed to estimate BVL from BL/FU pairs acquired with the same scanner and the same acquisition sequence. A detailed description is given in the following subsections. A schematic presentation is given in Fig. 1.

#### Preprocessing

Preprocessing (Fig. 1A) included reslicing to 1mm cubic voxels and (if necessary) rearranging to transversal orientation. Next, a 176 × 208 × 208 matrix covering the entire skull and brainstem down to the foramen magnum was cropped from the image. These steps were performed independently for BL and FU. Finally, BL and FU crop were rigidly registered in halfway space using ANTS [20].

#### Non-linear registration

A convolutional U-Net [24] following the architecture described in [18] was trained specifically for non-linear registration of preprocessed BL/FU pairs (Fig. 1B). The training subset of the development dataset was used for this purpose.

The unsupervised learning approach proposed by the VoxelMorph learning framework for deformable medical image registration was used [18]. In brief, the two crops from the BL/FU pair were concatenated and served as input to the U-Net. The output of the U-Net was the 3D tensor field characterizing the local deformations required to warp the BL crop to the FU crop (matrix size of the deformation field = 176 × 208 × 208 × 3). The weighted sum of the cross-correlation of the warped BL crop with the FU crop plus the spatial gradient of the deformation field (for regularization) was used as loss function. Further details are provided in the Supplementary material (“3D-CNN for non-linear registration: architecture and training”, Supplementary Figs. 1, 2) and in [18].

Data augmentation was performed on the fly during the training including left–right flipping and rotation (same for both crops) and adding Gaussian noise (separately for both crops). The direction of the non-linear registration was randomly interchanged (e.g., BL crop warped to FU crop or reversed). The training was performed for 350 epochs with a batch size 2 using the Adam optimizer with learning rate 0.0001. The training took 5 days (python 3.10, pytorch 2.0, GPU NVIDIA RTX A5000 24 GB memory).

#### Computation of brain volume loss

First, a custom 3D-CNN [23] was used to segment the brain parenchyma in the BL crop (Fig. 1C, supplementary section “3D-CNN for BPV and TIV segmentation: architecture and training”, Supplementary Fig. 3). This resulted in a binary BL parenchyma mask (1/0 inside/outside). The brain parenchyma volume (BPV) at BL (${\text{BPV}}_{\text{BL}}$) was obtained by counting the voxels in the BL mask.

Then, the U-Net for non-linear registration was used to compute the BL-to-FU deformation field. This was used to warp the BL parenchyma mask from the BL crop to the FU crop (Fig. 1D). The BPV at FU (${\text{BPV}}_{\text{FU}\leftarrow \text{BL}}$) was estimated from the warped mask by summing the voxel intensities of the warped mask across all voxels (including boundary voxels with intensity < 1 due to interpolation).

Then, the processing was reversed. More precisely, the FU-to-BL deformation field for the same BL/FU pair was estimated by the U-Net for non-linear registration and used to warp the parenchyma mask segmented in the FU crop to the BL crop. In this (“backward”) processing, the BPV at FU (${\text{BPV}}_{\text{FU}}$) was computed from the original mask, the BPV at BL (${\text{BPV}}_{\text{BL}\leftarrow \text{FU}}$) was estimated from the warped mask.

Then, the percentage brain volume change from BL to FU was estimated according to the following “forward” (fw) and “backward” (bw) formulas

1 $${\Delta \text{BPV}}_{\text{fw}}=100*\frac{{\text{BPV}}_{\text{FU}\leftarrow \text{BL}}-{\text{BPV}}_{\text{BL}}}{{\text{BPV}}_{\text{BL}}}$$

2 $${\Delta \text{BPV}}_{\text{bw}}=100*\frac{{\text{BPV}}_{\text{FU}}-{\text{BPV}}_{\text{BL}\leftarrow \text{FU}}}{{\text{BPV}}_{\text{BL}\leftarrow \text{FU}}}$$

In principle, both formulas should yield the same result. However, in order to increase robustness, particularly by reducing asymmetry-induced bias [25], the two estimates were averaged

3 $$\Delta \text{BPV}=0.5*( {\Delta \text{BPV}}_{\text{fw}}+{\Delta \text{BPV}}_{\text{bw}})$$

#### Correction for distortion effects

Pilot experiments had shown an association between the apparent BL-to-FU change of the total intracranial volume (TIV) and the severity of the distortion of BL and FU image relative to each other. This was the rationale for using the apparent TIV difference between BL and FU to correct $\Delta BPV$ for distortion effects.

The TIV was segmented using the same custom 3D-CNN that was used for segmentation of the brain parenchyma [23], separately for BL and FU. The BL TIV mask was warped to the FU crop and the FU TIV mask was warped to the BL crop using the same BL-to-FU and FU-to-BL deformation fields as for the brain parenchyma. The percentage TIV change from BL to FU was computed analogous to Eqs. (1)–(3) as

4 $$\Delta \text{TIV}=0.5*( {\Delta \text{TIV}}_{\text{fw}}+{\Delta \text{TIV}}_{\text{bw}})$$

Then, regression analysis was performed in the training dataset with $\Delta BPV$ as independent variable and $\Delta TIV$ as predictor:

5 $$\Delta \text{BPV} ={m}_{\text{TIV}}*\Delta \text{TIV}+{c}_{\text{TIV}}$$

**Fig. 2:**
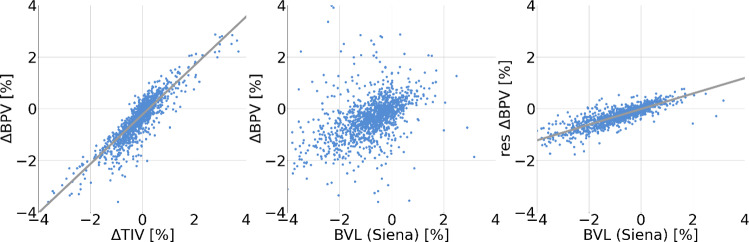
Distortion correction and rescaling. Linear regression of $\Delta BPV$ versus $\Delta TIV$ (left) in the training dataset was used for distortion correction, linear regression of the distortion-corrected residual of $\Delta BPV$ versus SIENA BVL (right) in the training dataset was used for rescaling. The scatter plot in the middle shows SIENA BVL versus $\Delta BPV$ (without distortion correction)

This resulted in ${m}_{TIV}$=0.95 (95%-confidence interval [0.93, 0.96]), ${c}_{TIV}$=-0.23 [-0.24,-0.22] (Fig. 2). In order to eliminate distortion effects from $\Delta BPV$, residuals were computed with respect to the regression line:

6 $$res\Delta \text{BPV} = \Delta \text{BPV}-{m}_{\text{TIV}}*\Delta \text{TIV}= \Delta \text{BPV}-0.95*\Delta \text{TIV}$$

The offset parameter ${c}_{TIV}$ in the regression model (5) represents the mean BVL in the training dataset in the absence of distortion effects ($\Delta \text{TIV}=0$) and, therefore, does not need to be subtracted from $\Delta BPV$ to correct for distortion effects (if ${c}_{\text{TIV}}$ would be included in formula (6), it would be removed by rescaling described below).

$\Delta \text{TIV}$ is computed for each new BL/FU pair and then used for “individual” distortion correction of $\Delta BPV$ according to formula (6).

The residuals can be assumed to be proportional to BVL, but they do not represent BVL in %. For rescaling, regression analysis was performed in the training dataset with the residual as dependent variable and the SIENA BVL estimate as predictor:

7 $$\text{res}\Delta \text{BPV}={m}_{\text{Siena}}*{\text{BVL}}_{\text{Siena}}+ {c}_{\text{Siena}}.$$

This revealed ${m}_{\text{Siena}}$= 0.30 (95% confidence interval [0.29, 0.31]), ${c}_{\text{Siena}}$= − 0.01 [− 0.02, − 0.01] (Fig. 2). The slope was used to rescale the residuals for quantitative estimation of the percentage BVL as

8 $$\text{BVL}=\text{res}\Delta \text{BPV}/{m}_{\text{Siena}} = \text{res}\Delta \text{BPV}/0.30.$$

The trained BrainLossNet is available from the authors upon request under a non-disclosure agreement for non-commercial use.

### Testing of BrainLossNet

#### Internal consistency

BrainLossNet computes the BVL as the mean of a forward and a backward estimate. More precisely, from Eqs. (8), (6), (4) and (3) one easily derives.

9 $$\text{BVL}=0.5*({\text{BVL}}_{\text{fw}}+ {\text{BVL}}_{\text{bw}})/0.30$$

with

10 $${\text{BVL}}_{\text{fw}/\text{bw}}=({\Delta \text{BPV}}_{\text{fw}/\text{bw}} - 0.95*{\Delta \text{TIV}}_{\text{fw}/\text{bw}})$$

Forward and backward estimates of BVL are independent in the sense that they are based on two different deformation fields. The consistency of forward and backward estimates was tested in the test sample of the development dataset using Bland–Altman analysis.

#### Agreement between BrainLossNet and SIENA

BVL estimates from BrainLossNet were compared with BVL estimates from SIENA using Bland–Altman analysis in the test datasets. Median and interquartile range (IQR) of the differences were computed as well as the intra-class correlation coefficient (ICC) of the BVL estimates.

#### Robustness to short-term test–retest variability of 3D-T1w-MRI

For the BL/FU pairs of consecutive back-to-back scans from the FTHP dataset, the true BVL is zero. Thus, the distribution of BVL estimates for these BL/FU pairs was used to characterize the robustness with respect to short-term test–retest variability of 3D-T1w-MRI, separately for BrainLossNet and SIENA. The width of the distribution of the BVL estimates was compared between BrainLossNet and SIENA using the Pitman test for comparison of variances of paired samples [26]. The Pitman test consists of testing for correlation between the sum and the difference between paired observations, a significant correlation indicating a significant difference regarding the variances.

## Results

The computation time for the complete BrainLossNet pipeline (including preprocessing) was approximately 150 s per BL/FU pair on a Linux workstation (CPU: Intel, Xeon, Silver, 4110 CPU 2.10 GHz 32 kernels, GPU Quadro P5000 16 GB). Computation time for SIENA depends on the resolution of the images. For an image pair with 260 × 256 × 256 voxels, the computation time was 63 min on the same workstation.

**Fig. 3:**
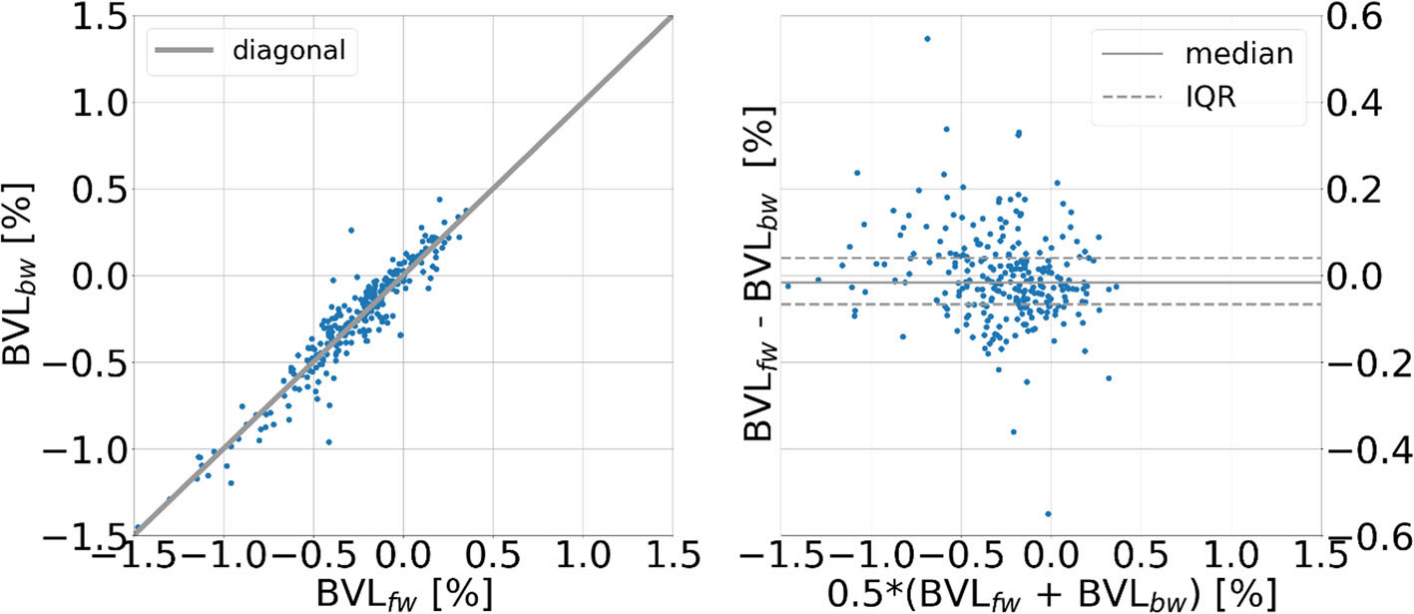
Consistency of forward and backward estimates. Scatter plot (left) and Bland–Altman plot (right) of forward versus backward BrainLossNet BVL estimates in the test sample from the development dataset. (IQR: interquartile range)

Concerning internal consistency of the BrainLossNet pipeline, Fig. 3 shows the scatter and the Bland–Altman plot of forward versus backward BVL estimates in the test sample from the development dataset. The median [IQR] forward–backward difference was − 0.02% [− 0.07%, 0.04%]. 95% of all forward–backward differences (absolute value) were smaller than 0.19%.

**Fig. 4:**
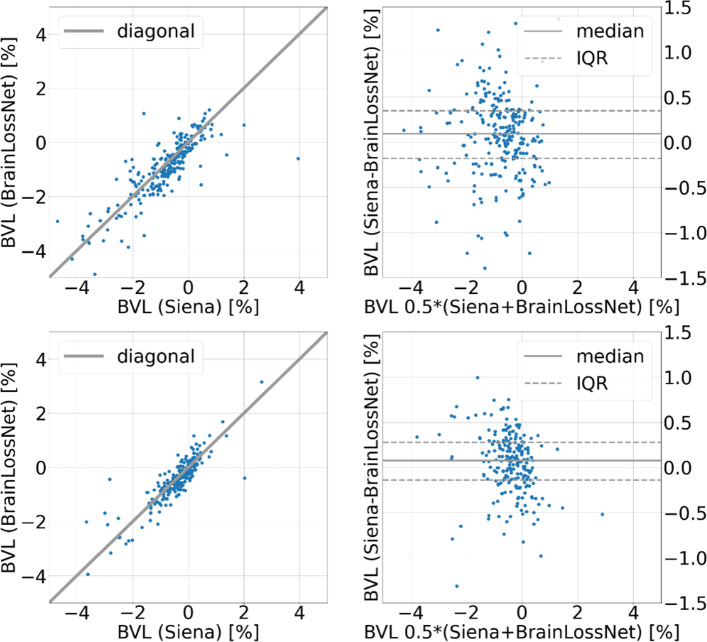
BrainLossNet versus SIENA. Scatter plot (left) and Bland–Altman plot (right) of BVL estimates from BrainLossNet versus SIENA in the test sample from the development dataset (first row) and in the MS dataset (second row). (IQR: interquartile range)

Concerning agreement between BrainLossNet and SIENA, Fig. 4 shows scatter and Bland–Altman plots of BrainLossNet versus SIENA BVL estimates. For the test sample from the development dataset, the median [IQR] SIENA-BrainLossNet BVL difference was 0.10% [− 0.18%, 0.35%]. The 95th percentile of the absolute differences was 1.23%, the ICC was 0.80. For the MS dataset, the corresponding values were 0.08% [− 0.14%, 0.28%], 0.67% (95th percentile), and 0.85, respectively.

**Fig. 5:**
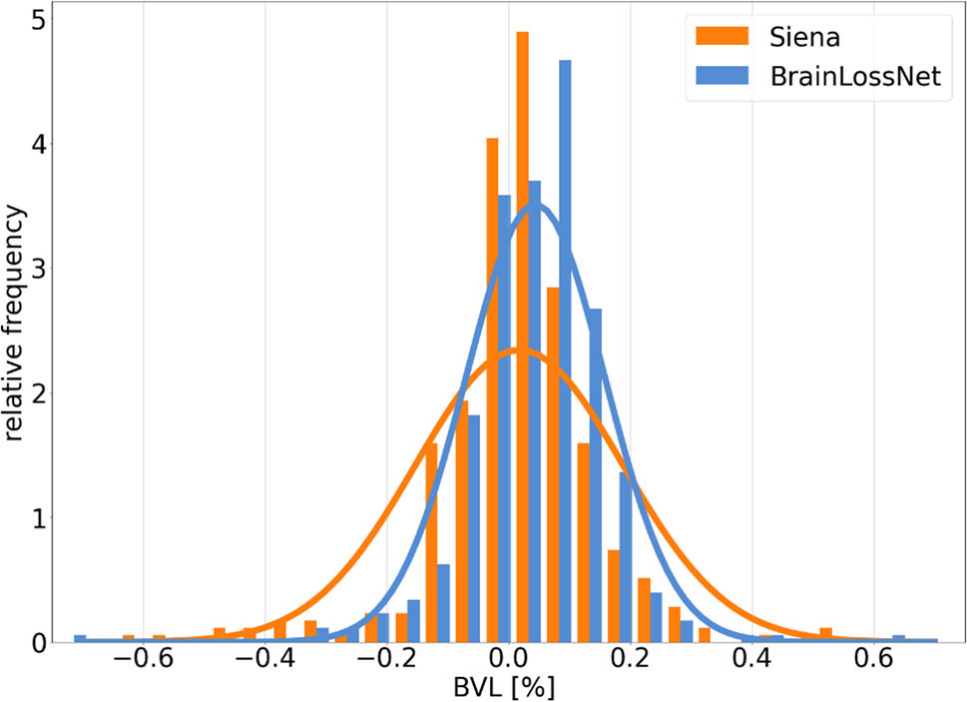
Robustness with respect to short-term test–retest variability of 3D-T1w-MRI. Histograms of the apparent BVL between consecutive back-to-back scans from the FTHP dataset. The continuous lines represent Gaussian fits

Concerning robustness with respect to short-term test–retest variability of 3D-T1w-MRI, Fig. 5 shows the histograms of the apparent BVL between consecutive back-to-back scans from the FTHP dataset. The mean apparent BVL was 0.02% ± 0.17% for SIENA and 0.04% ± 0.11% for BrainLossNet. The variance of the apparent BVLs was significantly larger for SIENA than for BrainLossNet (Pitman test: Spearman correlation coefficient 0.112, *p* = 0.036). The 95th percentile of the apparent BVL (absolute value) was 0.32% for SIENA and 0.20% for BrainLossNet.

## Discussion

BrainLossNet BVL estimation from a BL/FU pair took 2–3 min including all preprocessing steps. Most time was spent for the rigid BL-FU registration with the conventional ANTS method during preprocessing. Thus, further acceleration might be achieved by employing an additional CNN for this step. However, 2–3 min computation time of the current implementation makes BrainLossNet suitable for integration into routine workflows in clinical practice also without further acceleration.

The difference of BVL estimates between BrainLossNet and SIENA was not larger than 1.23% in 95% of the cases in the test sample from the development dataset and not larger than 0.67% in 95% of the cases in the MS dataset. This is in the same range as the 95th percentile of the within-patient variability of BVL estimates from short-term repeated MRI measurements with SIENA: 0.43%, 0.63%, and 1.15% in three different datasets [7]. Thus, BrainLossNet provided essentially the same BVL estimates as SIENA in the vast majority of cases, indicating that BrainLossNet and SIENA BVL estimates are largely equivalent.

The somewhat larger difference between BrainLossNet and SIENA in about 5% of the cases might be explained by better robustness of BrainLossNet to some extent. While the median difference of the BVL estimates from two consecutive scans in the FTHP dataset was similar for BrainLossNet and SIENA, the distribution of the differences was somewhat wider for SIENA (Fig. 5): the 95th percentile of the differences was 0.32% for SIENA compared to 0.20% for BrainLossNet. This suggests that among the about 5% cases with larger difference between BrainLossNet and SIENA, the BrainLossNet estimate might be more reliable.

In a pilot experiment, a strong association between $\Delta TIV$ and image distortions had been observed, in line with previous reports [15]. Based on the assumption that the strong correlation between ‘raw’ values of $\Delta BPV$ with $\Delta TIV$ observed here (Fig. 2) is mainly due to the fact that the impact of image distortion is similar for $\Delta BPV$ and $\Delta TIV$, the correlation was used to correct “raw” $\Delta BPV$ estimates for image distortions by computing residuals with respect to the regression line of $\Delta BPV$ versus $\Delta TIV$ in the training dataset. The proposed distortion-correction might have made BrainLossNet less sensitive to image distortions than SIENA. This might have contributed to its improved robustness.

For rescaling of the residuals to properly represent BVL in %, regression of the residuals versus SIENA BVL estimates was used. Importantly, the regression analyzes for distortion correction and rescaling were performed in the training dataset. The resulting formulas for distortion correction and rescaling were applied in all test datasets. No attempts were made to optimize distortion correction and/or rescaling for any of the test datasets. SIENA also involves a calibration step [5].

BrainLossNet relies on non-linear registration of BL/FU pairs, as do JI methods. However, in contrast to JI methods, which estimate brain volume change by integration of the Jacobian determinant of the deformation field, BrainLossNet applies the deformation field to binary brain masks obtained by delineation of the brain parenchyma with another CNN (Fig. 1). The rationale for this was that the computation of partial derivatives of the deformation field can be numerically challenging and, therefore, can result in noisy Jacobian determinant images (e.g., Fig. 7 in [22]). In pilot experiments comparing JI of the CNN-based deformation field with the warping of the binary parenchyma mask, the mask warping approach outperformed JI regarding agreement with Siena (Supplementary Fig. 4). We hypothesize that this is explained by local “non-smoothness” of the CNN-based deformation field. Conventional registration approaches usually ensure that the deformation field has certain smoothness properties, either by restricting the deformation field to a predefined space of functions (e.g., splines of a given order) or by the optimization process. As a consequence, the derivatives of the deformation field are well defined. In contrast, CNN-based non-linear registration does not employ strict smoothness constraints (beyond favoring smooth solutions by the loss function used for the CNN training).

Zhan and co-workers [27] recently proposed a different CNN-based method for BVL estimation. They used supervised training of an U-Net to reproduce SIENA change maps. Potential advantages of this approach compared to BrainLossNet include that it might be more robust when BL and FU scans were acquired with different MR scanners or different acquisition sequences. Potential advantages of BrainLossNet compared to the approach by Zhan and co-workers include that it might be more robust with respect to image distortions. Given that Zhan and co-workers used SIENA change maps for the U-Net training, the sensitivity to image distortions might be similar for their U-Net as for SIENA. Furthermore, BrainLossNet is easily adapted to estimate regional volume loss, which might be less straightforward for the U-Net method. These hypotheses should be tested in a head-to-head comparison of both methods.

BrainLossNet was trained and tested specifically to estimate BVL from BL/FU pairs acquired with the same scanner and the same acquisition sequence. Thus, its use should be restricted to this scenario (which is the most frequent scenario at many sites). We intentionally did not train BrainLossNet to estimate BVL also in scenarios with BL and FU from different scanners, because we hypothesized that this would have compromised BrainLossNet’s performance in BL/FU pairs acquired with the same MR scanner and the same acquisition sequence. There are no further restrictions on the use of BrainLossNet, in particular, there are no specific restrictions regarding the MR scanner or the 3D-T1w acquisition sequence. This is due to the fact that the development dataset used for training and testing of BrainLossNet was quite heterogeneous, essentially covering the whole range of image characteristics typically encountered in clinical practice.

In conclusion, BrainLossNet BVL estimates from BL/FU pairs acquired with the same MR scanner using the same acquisition sequence are essentially the same as those from SIENA in the vast majority of cases. In the remaining “outliers”, BrainLossNet estimates might be more reliable, given the better robustness of BrainLossNet. The BrainLossNet processing time of 2–3 min per case on a standard workstation makes it suitable for integration into routine workflows in clinical practice.

### Supplementary Information

Below is the link to the electronic supplementary material.

Supplementary file1 (DOCX 883 kb)
