# Supplementary material for: BrainLossNet: a fast, accurate and robust method to estimate brain volume loss from longitudinal MRI
Source: Int J Comput Assist Radiol Surg. 2024 Jun 16;19(9):1763–71. doi: 10.1007/s11548-024-03201-3 (PMC11365843; doi:10.1007/s11548-024-03201-3)
Supplement: Supplementary file 1 — Supplementary file1 (DOCX 883 kb) [file 11548_2024_3201_MOESM1_ESM.docx]

**Supplementary Material**

**BrainLossNet: a fast, accurate and robust method to estimate brain volume loss from longitudinal MRI**

Roland Opfer^a^ (ORCiD-ID 0000-0002-9911-5478), Julia Krüger^a^ , Thomas Buddenkotte^b^, Lothar Spies^a^, Finn Behrendt^c^, Sven Schippling^d,e^, Ralph Buchert^b^ (ORCiD-ID 0000-0002-0945-0724)

^a^jung diagnostics GmbH, Hamburg, Germany

^b^Department of Diagnostic and Interventional Radiology and Nuclear Medicine, University Medical Center Hamburg-Eppendorf, Hamburg, Germany

^c^Institute of Medical Technology and Intelligent Systems, Hamburg University of Technology, Hamburg, Germany

^d^Multimodal Imaging in Neuroimmunological Diseases (MINDS), University of Zurich, Zurich, Switzerland

^e^ Neuroscience and Rare Diseases (NRD), Roche Pharma Research and Early Development (pRED)

**Corresponding author:** Ralph Buchert, Department of Nuclear Medicine, University Medical Center Hamburg-Eppendorf, Martinistr. 52, 20246 Hamburg, Germany, Email: [r.buchert@uke.de](mailto:r.buchert@uke.de), Phone: +49 (0)40 7410-54347, Fax: +49 (0)40 7410-40265

**MR scanner models in the training dataset**

The 1525 BL/FU pairs in the training dataset had been acquired with the following scanner models: Siemens Skyra (3T, n=325), Siemens Avanto (1.5T, n=232), Philips Ingenia (1.5T, n=170), Siemens Aera (1.5T, n=148), Siemens MAGNETOM Vida (3T, n=91), GE DISCOVERY MR750 (3T, n=88), Siemens Symphony (1.5T, n=83), Siemens Verio (3T, n=46), Philips Achieva dStream (1.5T, n=38), GE Signa HDxt (1.5T, n=35), GE Signa HDxt (3T, n=29), Siemens Prisma (3T, n=27), Siemens HarmonyExpert (1T, n=26), Siemens Avanto_fit (1.5T, n=25), Siemens Espree (1.5T, n=23), Philips Intera (1.5T, n=21), GE DISCOVERY MR750w (3T, n=21), Siemens MAGNETOM Skyra (3T, n=16), Siemens MAGNETOM Lumina (3T, n=14), Siemens MAGNETOM ESSENZA (1.5T, n=13), Philips Ingenia S (1.5T, n=9), Siemens Avanto_DOT (1.5T, n=7), Philips Achieva (3T, n=7), Siemens Verio_DOT (3T, n=6), Philips Ingenia Ambition X (1.5T, n=6), Philips Panorama HFO (1T, n=5), Siemens MAGNETOM Sola (1.5T, n=4), Siemens SymphonyTim (1.5T, n=4), Siemens Amira (1.5T, n=3), GE OPTIMA MR360 (1.5T, n=1), Siemens MAGNETOM Aera (1.5T, n=1), Philips Ingenia (3T, n=1).

**3D-CNN for non-linear registration: architecture and training**

The architecture of the 3D-CNN for non-linear registration is shown in Supplementary Figure 1 below. It follows a fully convolutional encoder-decoder (U-net) architecture with 3D convolutions with 3x3x3 kernel size as suggested in [1]. The encoder reduces the spatial dimension in four steps from 176x208x208 to 11x13x13 voxels. The decoder uses convolution layers, followed by nearest-neighbour up-sampling. Feature concatenation is employed for the long-range connections between encoder and decoder. Leaky ReLU [2] with slope 0.2 was used as activation function in each layer.

For the training of the network, the sum of the normalized cross-correlation (NCC) between the warped baseline (BL) crop (= $\phi(BL crop)$, Supplementary Figure 1) and the follow-up (FU) crop plus the weighted mean gradient norm of the deformation field was used as loss function. The second term, that is, the sum of the gradient strengths, is a measure of the smoothness of the deformation field. The mean gradient norm was weighted by the factor 10 to balance between accuracy and smoothness (Supplementary Figure 2). The convergence over the 350 epochs is shown in Supplementary Figure 2, separately for the NCC, the mean gradient norm as well as their weighted sum (= overall loss).

**3D-CNN for BPV and TIV segmentation: architecture and training**

The architecture of the 3D-CNN for the segmentation of BPV and TIV is shown in Supplementary Figure 3. It follows a fully convolutional encoder-decoder (U-net-like) architecture with 3D convolutions with 3x3x3 kernel size. The encoder reduces the spatial feature map size four times (using convolution with stride 2) and doubles the feature map number with each reduction. Starting with 16 feature maps of size 128x128x128 in the first layer, this leads to 256 maps of size 8x8x8 in the last encoder layer. The decoder uses convolution layers, followed by nearest-neighbour up-sampling and deep supervision in three layers [3]. Feature concatenation is employed for the long-range connections between encoder and decoder. A leaky ReLU [2] is used as activation function in each layer. Due to the rather large patches of 160x160x160 voxels covering about 2/3 of the brain, batch size was set to 1. Therefore, instance normalization, a special case of group normalization, was used instead of batch normalization.

The ground truth for the TIV and the BPV was derived using a validated algorithm [4] based on the Statistical Parametric Mapping framework (version SPM12, <https://www.fil.ion.ucl.ac.uk/spm/software/spm12/>) [5]. The 3D-CNN was trained on 1,975 T1w-images of 1,975 different patients from 170 different MRI scanners. During training, data augmentation including simulation of random bias fields and statistical noise in addition to standard augmentation techniques (rotation, flipping) was used. More details are provided in [6].

**References to the supplementary material**

1. Guha Balakrishnan AZ, Mert R. Sabuncu, John Guttag, Adrian V. Dalca (2019) VoxelMorph: A Learning Framework for Deformable Medical Image Registration.

2. Maas AL, Hannun AY, Ng AY (2013) Rectifier nonlinearities improve neural network acoustic modelsProc icml. Citeseer, pp 3

3. Dou Q, Yu L, Chen H, Jin Y, Yang X, Qin J, Heng P-A (2017) 3D deeply supervised network for automated segmentation of volumetric medical images. Medical image analysis 41:40-54.

4. Malone IB, Leung KK, Clegg S, Barnes J, Whitwell JL, Ashburner J, Fox NC, Ridgway GR (2015) Accurate automatic estimation of total intracranial volume: a nuisance variable with less nuisance. Neuroimage 104:366-372. <https://doi.org/10.1016/j.neuroimage.2014.09.034>

5. Ashburner J, Friston KJ (2005) Unified segmentation. Neuroimage 26:839-851. <https://doi.org/10.1016/j.neuroimage.2005.02.018>

6. Opfer R, Krüger J, Spies L, Ostwaldt AC, Kitzler HH, Schippling S, Buchert R (2022) Automatic segmentation of the thalamus using a massively trained 3D convolutional neural network: higher sensitivity for the detection of reduced thalamus volume by improved inter-scanner stability. Eur Radiol. 10.1007/s00330-022-09170-y<https://doi.org/10.1007/s00330-022-09170-y>

**Supplementary Figures**

**
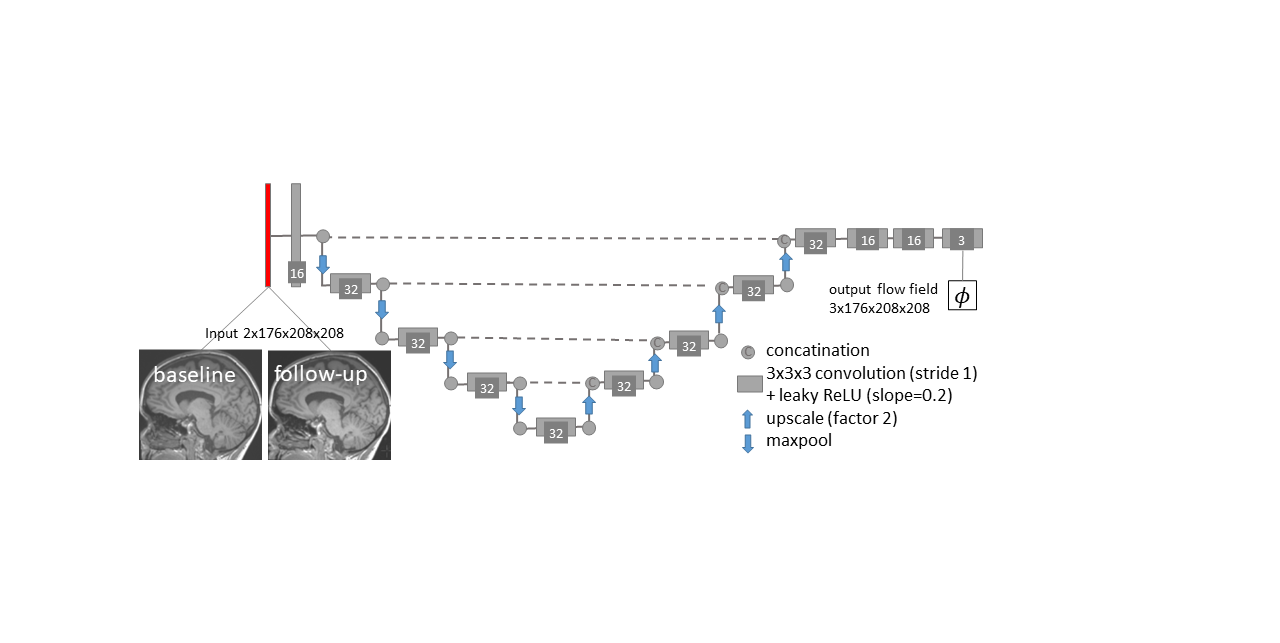
**

**Supplementary** **Fig. 1 3D-CNN for the non-linear registration.** Architecture of the 3D-CNN using 3D baseline (BL) and follow-up (FU) crops as input images and computing a 3D deformation field $\phi$ for non-linear registration of the BL crop to the FU crop

**
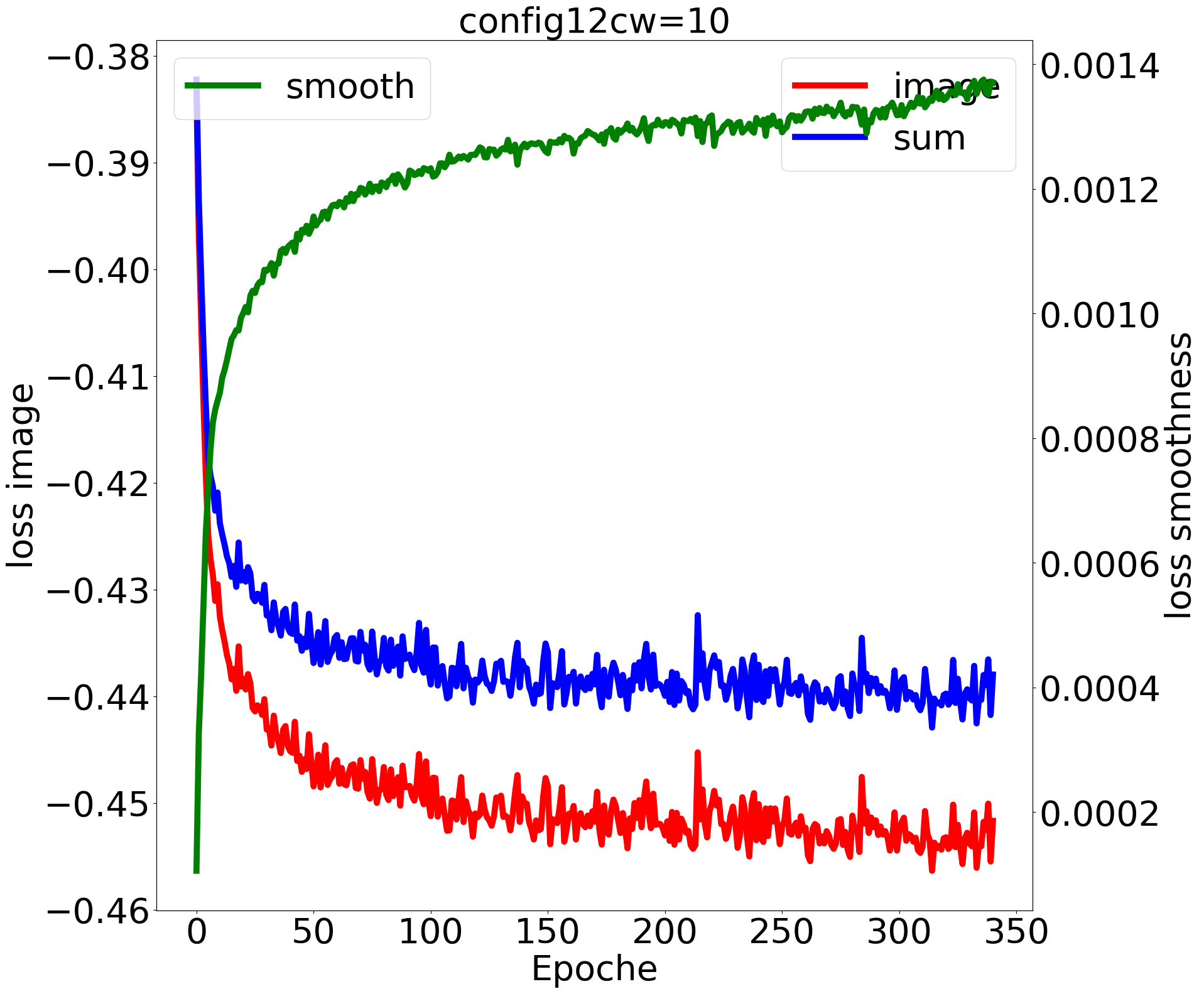
**

**Supplementary Fig. 2 Loss function during training of the 3D-CNN for non-linear registration.** The red curve (“loss image”, left axis) shows the (negative) normalized cross-correlation (NCC) between the warped BL crop and the FU crop, the green curve (“loss smoothness”, right axis) shows the unweighted mean gradient norm of the deformation field, and the blue curve (“sum”, left axis) depicts the overall loss (sum of “loss image” + 10 * “loss smoothness”)


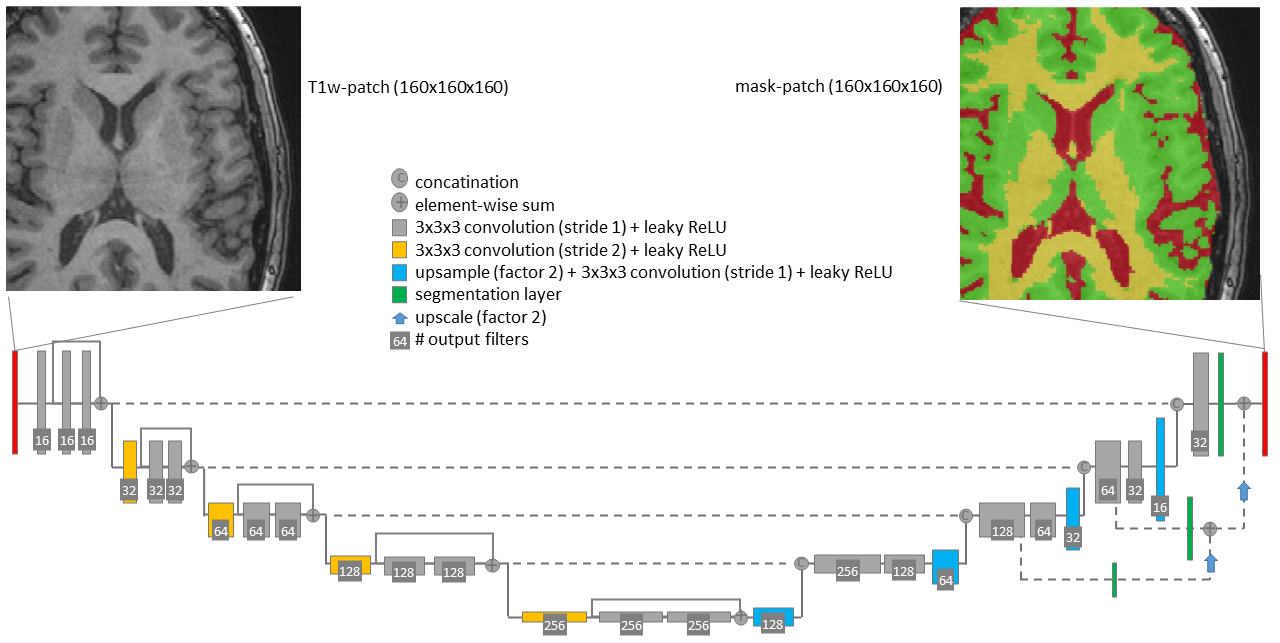


**Supplementary Fig. 3** **3D-CNN for the segmentation of BPV and TIV**

**
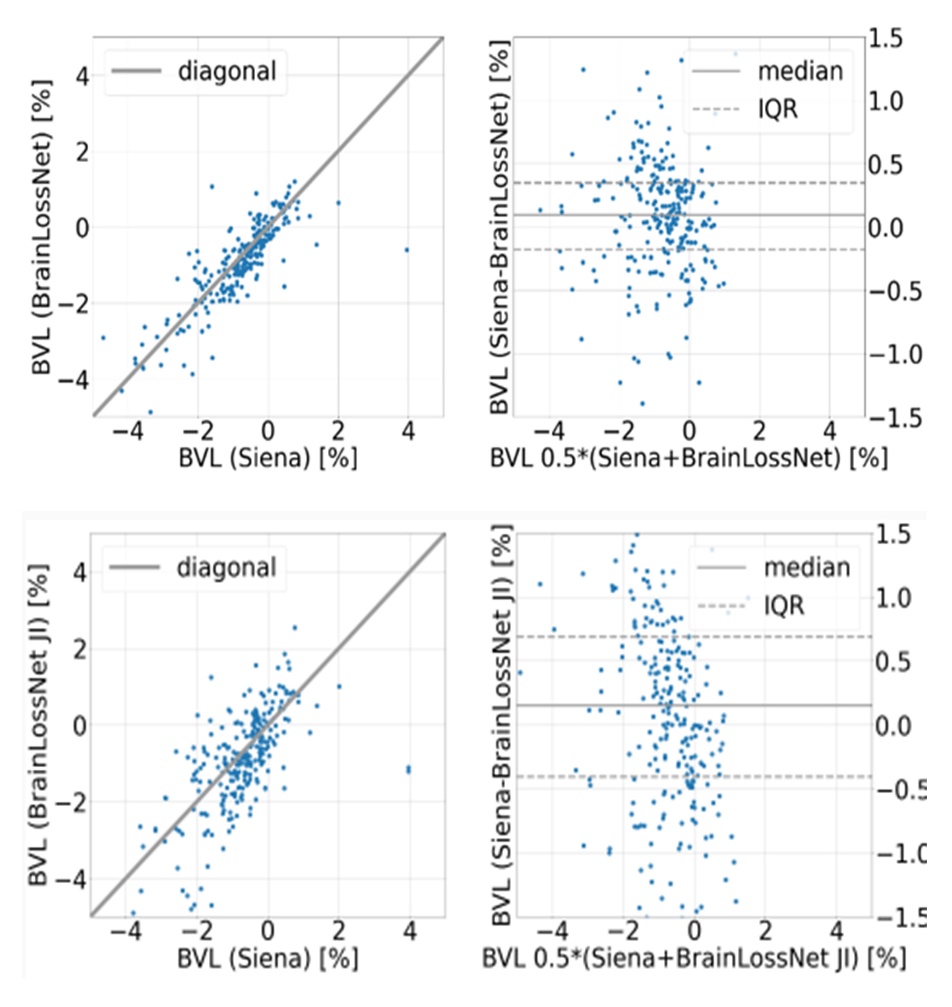
**

**Supplementary Fig. 4 Jacobian integration (JI) of the CNN-based deformation field for non-linear registration versus warping of binary parenchyma masks.** The top row shows the scatter plot (left) and the Bland-Altman plot (right) of the brain volume loss (BVL) estimates from BrainLossNet versus SIENA in the test sample from the development dataset (same as upper row in Figure 4 in the manuscript). The median [IQR] SIENA−BrainLossNet BVL difference was 0.10% [-0.18%,0.35%], and the 95th percentile of the absolute differences was 1.23% (as reported in the manuscript). The second row shows the results of the same experiment but using JI of the CNN-based deformation field instead of the proposed mask warping approach. The median [IQR] SIENA−JI BVL difference was 0.15% [-0.40%,0.69%], and the 95th percentile of the absolute differences was 1.96%.
